# Smart Antibiofilm Platforms Based on Synthetic Antimicrobial Peptides-Engineered Hydrogels

**DOI:** 10.3390/polym18040471

**Published:** 2026-02-12

**Authors:** Carpa Rahela, Bogyor Agota-Katalin, Butiuc-Keul Anca

**Affiliations:** 1 Department of Molecular Biology and Biotechnology, Faculty of Biology and Geology, Babeș-Bolyai University, 1 M. Kogalniceanu Street, 400084 Cluj-Napoca, Romania; rahela.carpa@ubbcluj.ro (C.R.); anca.keul@ubbcluj.ro (B.-K.A.); 2 Centre for Systems Biology, Biodiversity and Bioresources, Babeș-Bolyai University, 5–7 Clinicilor Street, 400006 Cluj-Napoca, Romania; 3 Doctoral School of Integrative Biology, Faculty of Biology and Geology, Babeș-Bolyai University, 1 M. Kogalniceanu Street, 400084 Cluj-Napoca, Romania

**Keywords:** antimicrobial peptides, AMP–hydrogel, biofilm

## Abstract

Chronic wounds and implanted medical devices remain highly vulnerable to biofilm-associated infections, which resist conventional antibiotics and immune clearance. Synthetic antimicrobial peptides (AMPs) have emerged as promising alternatives, offering tunable sequences, short lengths for cost-effective synthesis, and functional modifications that enhance stability and antibiofilm potency. Hydrogels provide an optimal delivery matrix by enabling localized AMP release, maintaining a moist wound environment, and supporting stimuli-responsive or sustained therapeutic action. This review highlights recent advances in peptide engineering strategies—including rational sequence design, chemical modifications, and self-assembling nanostructures—alongside hydrogel integration approaches ranging from physical entrapment to covalent tethering and infection-triggered release systems. Mechanistic insights into antibiofilm activity are discussed, supported by in vitro, ex vivo, and in vivo evaluation models. Beyond antimicrobial efficacy, multifunctional AMP–hydrogel systems can deliver complementary benefits such as hemostasis, anti-inflammation, or enzymatic biofilm dispersal, further accelerating tissue repair. Despite significant progress, translational challenges remain, including peptide stability, manufacturing costs, regulatory hurdles, and host safety. Future directions point toward AI-driven peptide design, programmable hydrogels, and point-of-care integration to realize safe, effective, and multifunctional AMP–hydrogel therapies for chronic wound management and biofilm eradication.

## 1. Introduction

Implanted medical devices and chronic wounds provide ideal environments for biofilm occurrence, causing a persistent and concerning clinical challenge.

Chronic wounds (like venous ulcers, diabetic foot ulcers and pressure sores) are usually characterized by persistent inflammation, reduced blood flow and recurrent bacterial exposure, which make them prone to biofilm formation [1,2,3]. Bacteria can straightforwardly bind to implanted medical devices (prosthetic joints, catheters, stents, pacemakers, etc.) [4].

The bacteria produce a protective extracellular matrix within the biofilm, consisting of polymeric substances (proteins, extracellular DNA and polysaccharides), which act as a physical and chemical barrier which hinders the effective penetration of antibiotics [5]. The dormant state of the bacteria in a biofilm are frequently in contributes to their resistance, shielding them from antibiotics that target actively dividing cells, making them resistant even to strong antibiotic treatment [6]. Together, these features explain the persistent, recalcitrant nature of biofilm infections in wounds and on devices.

Biofilms therefore show high tolerance to conventional antibiotics, tolerance based on their specific, structured matrix with a protective function, and their altered bacterial physiology. Their persistence impedes the healing of wounds [7] and increases the risk of systemic infection and even device failure, requiring debridement or replacement of the implanted device [8]. These elements clarify the progression from chronic wound pathophysiology to biofilms as a major impediment to healing, thereby motivating the introduction of synthetic antimicrobial peptides as a promising strategy to address this challenge.

Therefore, clinical management frequently relies on repeated debridement and device exchange rather than pharmacological eradication, underscoring an unmet therapeutic need. Due to failure of conventional antibiotic therapies in effectively passing thorough the structure of a biofilm, innovative therapeutic strategies that manage to disrupt the very biofilm formation are needed to ensure the healing of wounds. Recent developments in biomaterials, especially the use of antimicrobial compounds in wound dressings, have demonstrated encouraging outcomes in the fight against infections linked to biofilms [9,10,11,12]. Advanced wound dressings infused with biofilm-fighting components, including silver nanoparticles, antimicrobial peptides, and hydrogels, have been developed—antimicrobial biomaterials that help avert and manage infections [13]. They are not simple physical barriers, but also actively dismantle biofilm formation [6].

Synthetic antimicrobial peptides (AMPs) represent a new generation exhibiting several advantages:

- Synthetic design makes possible a fine tuning of structure and properties to improve their activity [14].
- Shorter peptides are less expensive to manufacture and can be created quickly and in large quantities [15].
- Chemical or structural changes (e.g., D-amino acids, lipidation, cyclization, or PEGylation) can provide resistance to proteases, increase the structure stability, and enhance antibiofilm properties [16,17].

In the context of biofilms, these features support both rapid bactericidal action and activity against metabolically diverse cells within structured matrices.

Synthetic AMPs are seen as a powerful tool surmounting the limits of antibiotics in tackling biofilm-associated infections.

AMPs are regarded as potential therapeutic agents because they offer dual benefits: they exhibit broad-spectrum activity and can be specifically engineered to target pathogens. They can be administered in a sustained, targeted fashion or released in reaction to triggers like pH, enzymes, or temperature variations that align with infection scenarios [14]. However, translating AMPs to wound care requires local concentration at the infection site and protection from inactivation—objectives that hydrogel systems can address.

Hydrogels act as delivery platforms in antimicrobial schemes, particularly for biofilm-infected wounds [18]. Their structure, rich in water and biocompatible, enables them to sustain a moist wound environment that promotes healing and effectiveness of antimicrobial agents [19]. Hydrogels can also concentrate high levels of peptides right at the infection site, minimizing possible toxicity [20,21].

This review centers on the incorporation of synthetic AMPs within hydrogels, highlighting antibiofilm effectiveness, design approaches, delivery systems, and translational questions.

## 2. Methods for Biofilm Evaluation

A range of quantitative and qualitative techniques can be used to evaluate biofilm growth and to determine how effective different treatments are [22].

### 2.1. In Vitro Biofilm Assays

Viable cell enumeration involves directly counting living cells or colony-forming units (CFU) to quantify viable organisms. The biofilm is homogenized, diluted, and plated on agar, with colonies incubated for 24–72 h and counted visually. From the aliquot volume and dilution factor, the mean cell number can be calculated, and the method can also be used to generate calibration curves by relating optical density (OD) to experimental cell counts. Although it requires no specialized equipment, the technique is labor-intensive and susceptible to counting variability, especially at high colony densities [22].

Flow cell counting was developed to automate biofilm measurements and reduce human bias. In this approach, a homogenized biofilm suspension passes through a narrow aperture, and cells are counted as they flow through. The method is simple and highly sensitive but works best with dilute samples—since the aperture can clog—and it cannot distinguish live from dead cells [23].

Flow cytometry offers an alternative to flow-based counting methods but is significantly more expensive. In this technique, cells pass through a narrow channel and can be detected using laser light scattering, absorbance, and fluorescence, providing cell counts and detailed information on cell size, surface characteristics, and metabolic state. Additional insights can be gained through staining or fluorescent tagging, including the use of green fluorescent protein (GFP) [24].

A range of microscopy techniques can be used for cell counting and visualizing biofilm architecture. Fluorescence microscopy enables visualization of cellular structures and live/dead cells, but requires costly equipment, filters, and dyes, and becomes increasingly difficult with dense, three-dimensional biofilms. More mature biofilms can be assessed with a Petroff–Hausser counting chamber, which provides an accurately defined volume for cell density measurements [25].

Confocal laser scanning microscopy (CLSM) produces high-resolution, high-contrast images by using a pinhole to capture light only from a precise focal plane, enabling 3D reconstruction of biofilms [26]. Multiple lasers can excite different fluorescent markers to increase the information obtained [27]. However, CLSM systems are expensive and require highly trained operators.

There are also a variety of indirect methods developed to measure biofilm development. Crystal violet (CV) staining is one of the most widely used assays for visualizing and quantifying biofilms. Although originally used to distinguish Gram-positive from Gram-negative bacteria—since only Gram-positive cells retain the dye after ethanol decolorization—CV can permeate both cell types and thus serves broadly in biofilm assays, though it cannot differentiate live from dead cells [28]. Importantly, CV staining quantifies total biofilm biomass, including microbial cells and extracellular polymeric substances, but does not provide information on cell viability or metabolic activity. CV is commonly paired with microtiter plate assays, an inexpensive and reproducible method for studying early-stage biofilms and determining metrics such as the minimum biofilm inhibitory concentration (MBIC) and minimum biofilm eradication concentration (MBEC). In this approach, bacteria are grown in 96-well plates, washed to remove planktonic cells, and the remaining biofilm is stained for biomass quantification [29]. These assays enable rapid, high-throughput screening but have a major limitation: drugs are often added before the biofilm forms, which can produce false positives if planktonic growth is inhibited rather than the biofilm itself [30,31].

Biofilm activity can be assessed not by dyes or colony counts but by measuring metabolic activity using tetrazolium salts. These salts are incubated with the biofilm for 1–3 h, where cellular enzymes reduce the colorless compound to a fluorescent formazan product that can be quantified by fluorescence spectroscopy, enabling real-time metabolic analysis [32]. It is important to note that tetrazolium-based assays are generally considered end-point measurements, as the accumulation of formazan products and prolonged exposure to the reagents can compromise cell integrity and interfere with biofilm structure. Consequently, while MTT and XTT assays provide valuable information on metabolic activity, they may not be suitable for repeated or longitudinal measurements of biofilm viability. In contrast, resazurin-based assays rely on the reversible reduction in resazurin to the fluorescent product resorufin and are generally considered non-destructive, allowing viability assessment without compromising cell integrity or biofilm structure, making them more suitable for repeated or continuous longitudinal measurements. Accordingly, resazurin-based assays overcome the examples of incompatibility presented and associated with tetrazolium salts, as they are non-toxic and do not require cell lysis, offering clear advantages in the assessment of biofilm viability [33].

Each biofilm analysis method has its own strengths and limitations, making results prone to methodological variability. Comparing findings across studies is challenging, especially because definitions of the MBEC differ—some authors describe it as the lowest concentration preventing visible growth, while others define it as the dose that eliminates 99.9% of the biofilm relative to controls [34].

### 2.2. Ex Vivo Models for Biofilm Assays

Chronic wounds represent a major global health and economic burden [35]. Biofilms represent a major contributor to infections (65%) and antibiotic failure, being involved in a wide range of infections, including medical devices and implants [36], chronic infections, lung, bladder, wound, dental, skin, ear, nose and throat, sinusitis, and orthopedic infections [37].

Animal models of wound healing in the presence of bacterial biofilms provide valuable insight into host responses and the impact of antimicrobial treatments [38]. However, they are costly, time-consuming, and unsuitable for high-throughput screening. They also lack the experimental control over microbial conditions, environment, nutrients, and sampling, that in vitro systems offer. Biofilms grown on different nonbiological surfaces can vary markedly, for example, those formed on glass differ significantly from those on steel plates [39]. Likewise, comparisons of biofilms on polystyrene microtiter plates with tissue-associated communities in zebrafish have revealed major differences in gene expression, indicating that biofilm formation alone represents only part of the bacterial response seen in real tissue environments [40]. Although in vitro models can generate robust biofilms, the dermal substrate itself may influence biofilm characteristics and alter responses to treatments. Using skin as the attachment surface and nutrient source would therefore produce biofilms that more closely resemble those found in wounds. A comprehensive review of existing in vivo and in vitro biofilm models is available elsewhere [41].

## 3. Peptide Engineering Strategies

**Figure 1:**
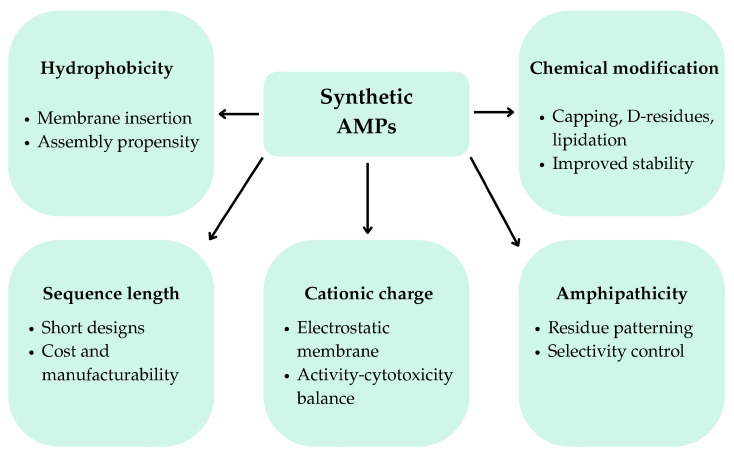
Peptide engineering strategies for synthetic AMPs. Key physicochemical parameters governing peptide engineering strategies that balance antibiofilm efficacy, stability, cytotoxicity, and compatibility with polymer-based delivery systems. AMPs, antimicrobial peptides.

Synthetic AMPs offer key advantages over natural AMPs because their sequences and parameters can be systematically adapted (e.g., charge, hydrophobicity, amphipathicity, or length) and/or chemically modified to improve stability, selectivity, and manufacturability while retaining membrane-active mechanisms that underpin antimicrobial function which is a key factor in combating antimicrobial resistance. Therefore, rational engineering of AMPs enables “fit-for-purpose” peptides with physicochemical properties that are matched to the infection microenvironment, while facilitating the downstream of formulation constraints [42]. In polymer-based delivery systems, the peptide design choices directly influence hydrogel loading efficiency, network interactions, and release kinetics, reinforcing the need for co-optimization of peptide and material properties. The key physicochemical parameters and engineering strategies governing synthetic AMP design are summarized schematically in Figure 1.

Synthetic AMPs are commonly engineered by systematically adjusting net positive charge, hydrophobicity, and amphipathicity. These three interdependent parameters govern electrostatic attraction to anionic bacterial envelopes, membrane insertion, and selective killing over mammalian cells. In α-helical scaffolds, optimizing helicity and the segregation of polar/cationic versus hydrophobic residues can enhance membrane disruption while limiting host toxicity, but changes in any one variable often propagate to the others and therefore require iterative optimization rather than single-parameter tuning [42]. A practical illustration of design-rule implementation is provided by a study that constructed a panel of nine-residue peptides engineered to reduce synthesis cost while constraining hydrophobic residue fraction (30–60%), net charge (+4 or +6 via Lys/Arg), and residue patterning (center-symmetric layouts and inclusion of WW/WWW motifs), achieving an optimized balance between antibacterial potency and cytotoxicity [43]. Complementing this strategy, another study developed a rational framework using libraries of Trp/Arg-rich engineered peptides, identifying minimal effective lengths (down to 16 residues) and specific Trp positional arrangements as key determinants of antibacterial potency while preserving low mammalian cell toxicity—demonstrating that residue placement can critically influence functional performance [44]. More generally, targeted amino-acid substitution (single or multiple residues) is widely used to refine physicochemical profiles (hydrophobicity, amphipathicity, and charge distribution) and can yield potent derivatives even when only a small subset of the parent sequence is responsible for activity [45]. Altogether, these approaches emphasize that antibiofilm performance is not dictated solely by charge or hydrophobicity, but by their spatial organization and the secondary structure propensity they impose under physiological conditions [42,45]. While charge and hydrophobicity are central to all AMP designs, their optimal ranges and spatial organization differ between planktonic antibacterial activity, antibiofilm efficacy, and compatibility with polymer-based delivery systems. Consistent with these principles, it was reported that effective synthetic antibiofilm AMPs typically fall within balanced ranges of length, net charge, and hydrophobicity, reinforcing the importance of balanced physicochemical design rather than maximal membrane activity [46]. The principal peptide engineering strategies and their general effects on biofilm-related outcomes, stability, and translational considerations are summarized in Table 1. Recently, computational and machine-learning–assisted design approaches have been increasingly applied to accelerate synthetic AMP discovery by navigating multidimensional sequence–property relationships and prioritizing candidates that balance antibiofilm activity, cytotoxicity, and stability prior to experimental validation [45,47].

Clinical translation of antibiofilm AMPs is frequently limited by proteolysis and off-target membrane damage. Consequently, engineering strategies often prioritize stability and selectivity alongside activity. Truncation is routinely used to reduce production costs and can preserve or even improve activity when nonessential residues are removed, provided amphipathicity and charge are retained [42]. Stereochemical engineering via D-amino acid substitution is a particularly direct route to protease resistance: incorporation of D-residues reverses stereochemistry and can enhance stability while largely maintaining net charge and overall hydrophobicity, although amphipathicity and local structure may change [42]. A clinically oriented illustration of this concept reported on the D-amino acid form of a branched peptide (SET-M33D), emphasizing improved protease tolerance and broad antibacterial activity against clinically relevant pathogens. In addition, the use of non-natural amino acids and peptidomimetic design expands accessible chemical space and can improve stability, bioavailability, and target selectivity relative to linear peptides composed exclusively of proteinogenic residues [45]. Importantly, antibiofilm efficacy must still be validated in relevant models. Representative examples of engineered antimicrobial peptides and their reported effects on biofilm-related outcomes are provided in Table 2. For example, WLBU2 (RRWVRRVRRVWRRVVRVVRRWVRR) is a synthetic cationic antimicrobial with a shown antibiofilm activity [48]. This peptide has subsequently been demonstrated in vivo, where WLBU2 significantly inhibited *Pseudomonas aeruginosa* biofilm formation in a murine catheter-associated infection model at sub-lethal concentrations without inducing hemolysis [49]. Mechanistically, WLBU2 exerts its antibiofilm activity primarily through rapid membrane destabilization driven by its high cationic charge and amphipathic α-helical structure, leading to increased membrane permeability and bacterial cell damage. In addition to direct membrane disruption, WLBU2 was shown to significantly downregulate key quorum-sensing genes (*lasI*, *lasR*, *rhlI*, and *rhlR*) in *P. aeruginosa* at sub-MIC levels [28], while in vivo antibiofilm efficacy and lack of hemolysis were demonstrated in a murine catheter model [49].

From a formulation and translational perspective, stabilization strategies (including chemical modification and conjugation approaches) are frequently paired with pharmaceutical development considerations, reinforcing that sequence engineering and delivery strategy should be co-designed rather than treated independently [50].

**Table 1:** Peptide engineering strategies governing antibiofilm activity, stability, and translational suitability.

Engineering Strategy	Design Purpose	Effect on Biofilm	Effect on Stability or Cytotoxicity	Reference
Net cationic charge	Promote electrostatic attraction to anionic bacterial membranes and extracellular polymeric substance (EPS)	Increased bacterial binding and membrane disruption	Excessive charge may increase hemolysis	[42,45]
Amphipathicity and residue patterning	Enable membrane insertion while preserving selectivity	Enhanced killing of planktonic and biofilm-embedded bacteria	Poor patterning can reduce selectivity	[43]
Sequence shortening	Reduce synthesis cost and manufacturing burden	Activity preserved if key residues retained	May reduce stability unless compensated	[43,44]
D-amino acid substitution	Prevent protease recognition	Maintained antibiofilm activity in protease-rich environments	Strongly improves stability; minimal toxicity change	[42]
Non-natural residues/peptidomimetics	Expand chemical space and backbone diversity	Preserved membrane activity with enhanced durability	Improves half-life; may complicate synthesis	[45]
Terminal capping	Protect against exopeptidases and stabilize helices	Often increases potency	Improves stability; modest toxicity risk	[51]
Lipidation	Increase membrane affinity and local concentration	Enhanced antibiofilm potency	can increase cytotoxicity if over-hydrophobic	[45]
Self-assembly	Increase local peptide density; multivalency	Strong antibiofilm effects via micellization	Improves stability; activity context-dependent	[43,52]
Iterative multi-parameter optimization	Simultaneously balance activity, selectivity, and stability	Improved efficacy across planktonic and biofilm states	Reduces toxicity by avoiding over-optimization of single parameters	[46]
Polymer-aware peptide design	Enable efficient integration into hydrogels and polymer matrices	Sustained local activity via improved retention and release control	Improves functional persistence; minimizes systemic exposure	[18]

The effects summarized reflect the general trends reported across multiple studies. Antibiofilm efficacy, stability, and cytotoxicity are strongly context-dependent and influenced by peptide sequence, concentration, target organism, and experimental models.

**Table 2:** Representative examples of peptide engineering strategies and their reported effects on biofilm-related outcomes.

Peptide/Strategy	Engineering Approach	Key Antibiofilm Outcome	Notable Limitation	Reference
WLBU2	Charge optimization; synthetic sequence	Reduced *P. aeruginosa* catheter biofilms in vivo; QS inhibition	Requires formulation for local delivery	[49]
K6	Ultrashort, Trp-rich, self-assembling	Micelle-mediated biofilm clearance in mixed infections	Activity depends on assembly state	[43]
GL13K	Self-assembly on surfaces	Reduced *Staphylococcus aureus* viability on titanium	Modest effect magnitude	[52]
Stapled AMPS	Helix stabilization	Increased protease resistance	Increased hemolysis risk	[51]
Lipidated AMPS	Fatty-acid conjugation	Enhanced membrane activity	Cytotoxicity risk	[45]
Polymer–Peptide Hybrid AMPs	Chemical conjugation or covalent incorporation into polymer matrices	Sustained antibiofilm activity with improved material compatibility	Design complexity; requires co-optimization of peptide and polymer	[18]

Terminal protection and side-chain functionalization are widely employed to increase serum stability, tune helicity, and improve activity in complex biological matrices. N-terminal acetylation and C-terminal amidation are among the most prevalent modifications; they reduce susceptibility to exopeptidases and can increase conformational stability and antimicrobial potency [51]. Mechanistically, capping can favor helix nucleation and stabilize amphipathic helices at membrane interfaces, features that are often linked to stronger activity against bacteria relative to uncapped analogs [51]. Consistent with this, amidation and other N-terminal modifications (e.g., acetylation, methylation) are described as strategies that can enhance resistance to enzymatic degradation and improve activity under physiological conditions [45]. Lipidation (fatty-acid conjugation) is another frequently used approach that strengthens peptide–membrane interactions and is discussed to increase potency, protease resistance, and, in some contexts, reduce cytotoxicity by modulating membrane affinity and peptide assembly behavior [45]. At the same time, patent- and literature-level surveys of antibiofilm AMPs highlight the broad use of chemical handles (e.g., acyl chains, PEGylation, and terminal acetylation) as formulation-oriented modifications intended to improve stability and performance against biofilms [53]. While hydrocarbon stapling is primarily discussed as a general strategy to constrain peptides and stabilize bioactive conformations, its relevance in AMP optimization lies in enforcing helical structure and potentially improving cell/biofilm penetration, objectives aligned with capping and other helix-stabilizing chemistries [51].

An emerging design paradigm is to engineer peptides that self-assemble into supramolecular structures (e.g., micelles, nanoribbons, nanofibers), thereby increasing local peptide concentration, enhancing stability, and enabling multivalent membrane interactions [45]. Within the micelle-forming peptide class, one study demonstrated that a nine-residue lead peptide (K6) self-assembled into nanostructured micelles in PBS (average diameter ~174 ± 48.82 nm), and this micellization correlated with strong antibiofilm activity against *P. aeruginosa* and *S. aureus*, including effective clearance of mixed-species biofilms in a mouse persistent infection model [43]. Their design explicitly combined hydrophobic WW/WWW motifs with terminal Lys clusters to promote cation–π and hydrophobic interactions supporting assembly while maintaining a cationic surface [43]. In parallel, self-assembly has been leveraged to improve peptide retention on biomaterial surfaces; GL13K, a cationic amphipathic peptide, can self-assemble (e.g., nanoribbons under conditions that partially neutralize charge), and self-assembled states adopting α-helical or β-sheet secondary structures formed more effective antimicrobial titanium coatings than unordered GL13K, underscoring that both supramolecular organization and secondary structure can be determinative for activity in interface-driven applications [52]. More broadly, self-assembling AMPs are discussed as offering improved stability across physiological conditions and reduced proteolytic degradation, features attractive for antibiofilm deployment in wound and device contexts [45]. It is also important to note that in contrast to planktonic targeting, antibiofilm peptide design must also account for penetration of the EPS matrix, which can necessitate distinct charge density, amphipathic balance, or supramolecular organization to overcome diffusion barriers and local peptide sequestration [45,53].

Successful incorporation of engineered AMPs into hydrogel matrices requires reconciling potent antibiofilm mechanisms with stability in protease-rich environments, acceptable host compatibility, and scalable synthesis. Reviews of AMP development for biofilm and polymicrobial infections consistently identify degradation by serum proteases, potential cytotoxicity, short half-life, and high production cost as principal barriers, motivating the use of sequence optimization, non-natural residues, self-assembly and lipopeptide strategies, and biomaterial-based delivery to improve exposure at the infection site while limiting systemic liabilities [53]. In this context, “design-for-formulation” choices, such as shortening sequences to reduce cost, applying terminal capping to extend lifetime, or engineering self-assembly to enhance local concentration, are directly aligned with the requirements of hydrogel delivery systems that aim to provide localized, sustained, and safer antibiofilm therapy [43,51]. Ultrashort peptide length (9 residues) has been highlighted as an effective cost-reduction strategy while preserving strong antibiofilm activity, complementing findings from another study showing that minimum effective lengths of approximately 16 residues can be maintained when critical residue positioning and amphipathic organization are preserved [43,44].

Collectively, antibiofilm peptide engineering relies on precise control of amphipathicity, cationic charge, and hydrophobic balance, complemented by sequence minimization, stereochemical modification, and chemical functionalization to improve stability, protease resistance, and selectivity. Emerging strategies incorporating self-assembly and formulation-aware designs further enhance antibiofilm efficacy while addressing translational constraints such as cytotoxicity, durability, and manufacturability, supporting effective integration into hydrogel-based delivery systems.

## 4. Antibiofilm Activity of AMPs

### Mechanism of Action

Biofilms are structured bacterial communities held together by EPS produced by the bacteria themselves, typically consisting of polysaccharides, proteins, and extracellular DNA [54]. Despite their wide applications in agriculture [55] and environmental remediation [56], biofilms possess serious problems in industrial and clinical environments. Biofilms protect bacterial cells from a wide range of harmful environmental stresses and generate persistent cell populations that can survive stress exposure, later regrowing once environmental conditions become favorable. In clinical settings, biofilms make a major contribution to chronic and recurrent infections [57].

Biofilms are formed through sequential stages of adhesion, growth with matrix production, maturation, and eventual dispersion [58]. During this process, cells become firmly anchored and significantly more resistant to antimicrobials and host defenses, displaying slow growth, matrix entrapment, and the ability to enter dormant states. Consequently, bacteria embedded within biofilms are believed to be 10–1000 times more tolerant to conventional antibiotics compared with planktonic ones [59,60]. This increased tolerance is particularly problematic when biofilms develop on the surfaces of medical devices, including implanted instruments, where they can lead to serious and difficult to treat healthcare-associated complications [61,62].

Biofilms exhibit strong antimicrobial resistance by blocking or neutralizing drugs through mechanisms such as efflux pumps, antibiotic-sequestering matrix components, and enzymatic degradation, allowing bacteria to grow even at concentrations normally inhibitory to planktonic cells. Biofilms also show high tolerance, enabling bacterial cells to survive, without growing or dying, in the presence of bactericidal agents through slow growth, persisted cells, and stress-response mechanisms that prevent downstream antibiotic toxicity. The mechanisms of biofilm resistance and tolerance have been extensively discussed in previous work [60].

Consequently, preventing biofilm formation or disrupting established biofilms has become an attractive strategy for combating chronic bacterial infections [63,64]. There is growing interest in developing novel microbial control approaches, and in recent years substantial efforts focused on creating safe, rapid, and effective antibiofilm technologies. Antimicrobial peptides (AMPs), ancient components of innate immunity found across all kingdoms of life, have shown broad antimicrobial activity and potential to combat drug-resistant pathogens [65]. Increasing attention has been directed toward their antibiofilm properties, and many AMPs have demonstrated the ability to prevent or disrupt biofilms through mechanisms such as inhibiting adhesion, increasing motility gene expression, reducing matrix synthesis, or directly killing bacterial cells [66,67,68,69]. Despite this potential, optimization of AMPs for antibiofilm use remains at an early stage. Several AMP databases exist [70,71,72,73], focused exclusively on peptides evaluated against planktonic cells. To address this gap, the BaAMPs database was developed to comprehensively gather information on AMPs specifically tested against microbial biofilms [74]. BaAMPs include peptide sequences and physicochemical properties, detailed antibiofilm activity data, experimental conditions and evaluation methods, microbial species/strains examined, and is freely accessible online at http://www.baamps.it (accessed on 28 November 2025).

**Figure 2:**
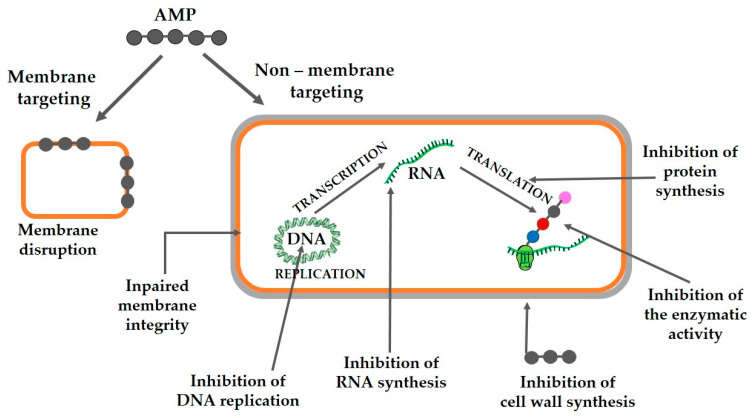
Mechanisms of AMPs’ action. AMPs act by membrane disruption or by targeted inhibition of critical bacterial pathways such as cell wall synthesis, DNA replication, RNA synthesis, protein synthesis and enzymes activity.

Antimicrobial peptides typically contain 10–60 amino acids [75], and their amphipathic nature is central to their ability to integrate into membranes or enter the cytosol [76,77] interfering with several cellular mechanisms as shown in Figure 2. Their mechanisms depend on charge, structure, length, concentration, hydrophobicity, and membrane composition [78]. AMPs can destroy bacteria by breaking the cell wall and membrane, by intracellular action, through a combination of dual destruction mechanisms, and by acting on the bacterial biofilm [79]. Many peptides act by altering intracellular functions, and in that way, they kill bacteria, as for example, the inhibition of DNA, RNA, and protein synthesis [80,81,82]. Different studies showed that most AMPs share a combination of these mechanisms [79,80,82,83].

**Figure 3:**
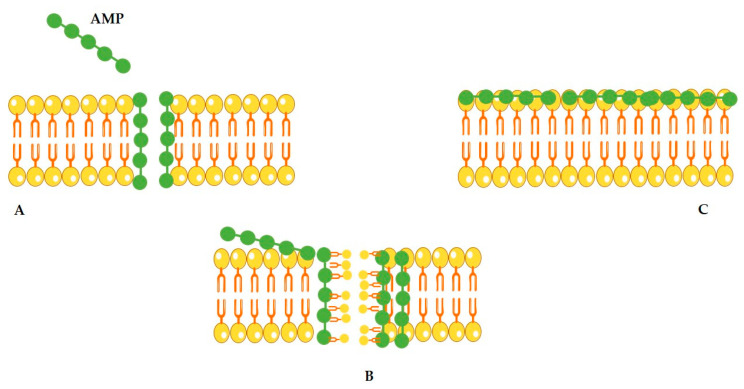
Models proposed to explain the mechanism of action of amphipathic α-helix AMPs. (**A**) Barrel-stave pore model; (**B**) toroidal pore model; (**C**) carpet model.

Antimicrobial peptides (AMPs) act through mechanisms distinct from conventional antibiotics [84,85]. Their effectiveness depends on their interaction with microbial membranes. Many AMPs disrupt bacterial inner or outer membranes, leading to cell death [86,87,88]. Membrane damage may result from the AMP binding to negatively charged surfaces, inhibition of protein, DNA or RNA synthesis, or interactions with specific intracellular targets [86,88,89]. Crucial to this process are electrostatic attractions between cationic AMPs and negatively charged bacterial membranes [90,91] which are rich in phospholipids in both Gram-positive and Gram-negative bacteria. For amphipathic α-helical peptides, several mechanisms have been proposed as reviewed by [92]. One is the barrel-stave pore model in which the α-helical peptides insert into the membrane and assemble into barrel-like aggregates, forming transmembrane, water-permeable pores [89,93]. Another model is the toroidal pore model, where peptides induce the formation of a transmembrane water-permeable pore, resulting in the association of several AMP molecules with lipid heads [89]. The carpet model proposes that AMPs bind to phospholipid headgroups and distribute themselves across the membrane surface in a carpet-like manner. Once a critical peptide concentration is reached, the membrane destabilizes and ultimately disintegrates into micelles [89]. These proposed models of amphipathic α-helix AMPs’ action are illustrated in Figure 3.

AMPs can interfere with biofilms at every stage of their development. They may prevent biofilm formation by disrupting bacterial signaling pathways, including triggering the production of guanosine tetraphosphate (ppGpp) and pentaphosphate (pppGpp) under nutrient-limited conditions, which suppresses nucleic acid synthesis. They can also reduce the expression of transport and binding protein genes essential for biofilm establishment. In addition, AMPs can disrupt mature biofilms by altering the membrane potential of bacterial cells [79,83].

Several AMPs also have activities on fungal cells. Antifungal peptides (AFPs) are emerging as promising therapeutic agents against fungal infections [69,94]. Most AFPs act through membrane-associated mechanisms, taking advantage of unique fungal membrane features—such as distinct sphingolipids, phosphatidylinositol content, and ergosterol—to target components like glucosylceramides, mannosyldiinositol phosphorylceramide, or specific fungal proteins, which enhances selectivity and reduces resistance development [95]. AFPs can be categorized by structure, mechanism, or origin, into natural, semisynthetic, and synthetic peptides [96]. Here we referred to the antifungal activity of semisynthetic or synthetic peptides. Semisynthetic and synthetic antifungal peptides are engineered to enhance pharmacological performance while minimizing immunogenicity and other adverse effects associated with natural peptides [96]. Their antifungal activity is determined by biophysical features—including net charge, stereospecificity, hydrophobicity, secondary structure, peptide length, and amphipathicity—with increases in positive charge, hydrophobicity, or amphipathicity, generally improving their efficacy [96,97].

## 5. Hydrogel Integration and Triggered Release

**Figure 4:**
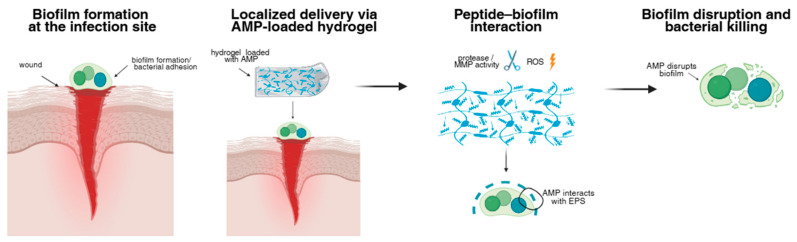
Schematic overview of AMP-loaded hydrogel application for biofilm-associated infections. Following biofilm formation at the infection site, AMP-loaded hydrogels enable localized peptide delivery and retention. Peptides interact with the biofilm EPS matrix through a combination of diffusion, contact-mediated activity, and infection-triggered release mechanisms. Elevated protease or matrix metalloproteinase (MMP) activity and reactive oxygen species (ROS) characteristic of infected tissues can promote peptide release, leading to biofilm disruption and bacterial killing while minimizing systemic exposure. AMP, antimicrobial peptide; EPS, extracellular polymeric substance; MMP, matrix metalloproteinase; ROS, reactive oxygen species.

Hydrogels are three-dimensional, crosslinked polymer networks capable of retaining large amounts of water while maintaining structural integrity. Hydrogels have emerged as one of the most versatile and clinically relevant platforms for the localized delivery of AMPs in the context of chronic wounds, implanted medical devices, and biofilm-associated infections caused by pathogens such as *S. aureus* or *P. aeruginosa*. Their mechanical properties, three-dimensional structure and high hydration provide a biomimetic environment that supports tissue compatibility while enabling controlled incorporation and presentation of therapeutic agents. Unlike conventional topical formulations or systemic delivery, hydrogel-based systems can maintain high local peptide concentrations at the infection site, reduce systemic exposure, and protect labile peptides from rapid dilution or clearance [98,99]. As a result, hydrogels are increasingly recognized as enabling matrices that bridge the gap between potent AMP bioactivity and the hostile biochemical conditions encountered in infected tissues, by sustaining local peptide exposure and reducing subtherapeutic concentration gradients that promote resistance. A schematic representation of an AMP-loaded hydrogel application for localized biofilm disruption is shown in Figure 4.

AMPs can be integrated into hydrogels through several complementary strategies, most commonly physical entrapment within the polymer network, covalent tethering to the hydrogel backbone or surface, or hybrid approaches combining both immobilized and releasable peptide fractions [100]. The main hydrogel integration strategies for AMP delivery, together with their characteristic mechanisms, advantages, and design limitations, are summarized in Table 3. Natural polymer hydrogels (including alginate, chitosan, gelatin, collagen, and hyaluronic acid) are frequently used due to their inherent biocompatibility, biodegradability, and resemblance to native extracellular matrix components [39,99]. Synthetic systems such as polyethylene glycol (PEG), polyacrylamide, and GelMA offer greater control over mechanical strength, crosslinking density and functionalization chemistry, enabling precise adjustment of peptide loading, orientation, and release kinetics [99,100]. The selection of hydrogel composition therefore plays a central role in determining AMP stability, spatial distribution, and interaction with both host tissue and microbial biofilms.

Physical entrapment of AMPs within hydrogel matrices represents the simplest and most widely explored delivery approach. In these systems, peptides are loaded during gel formation and subsequently released through diffusion and/or hydrogel degradation. This provides sustained local exposure that can suppress planktonic bacteria and early-stage biofilm formation [100]. Entrapment-based systems are particularly attractive for wound dressings, where continuous peptide release can complement the moist environment required for healing [98]. However, several limitations have been consistently reported. Freely diffusing peptides remain susceptible to proteolytic degradation in chronic wound fluid, which is rich in host- and bacteria-derived proteases, leading to rapid loss of activity [98]. In addition, initial burst release is frequently observed during early hydrogel swelling, resulting in transiently high concentrations followed by subtherapeutic levels that may be insufficient to eradicate mature biofilms [99,100]. These disadvantages have guided the exploration of alternative strategies that improve peptide retention and durability.

Covalent tethering of AMPs to hydrogel networks offers a fundamentally different mode of action by immobilizing peptides at the material interface while preserving antimicrobial function. Using chemical methods such as amide coupling, thiolene reactions, click chemistry, or photoreactive crosslinkers, peptides can be anchored to polymer chains in a defined orientation and density [100,101,102,103]. Immobilized AMPs act primarily through contact-mediated killing, in which bacterial cells contacting the hydrogel surface experience membrane disruption without requiring peptide diffusion [103]. A compelling demonstration of this approach was reported by a research team that developed a photocrosslinked hydrogel functionalized with the synthetic antibiofilm peptide SAAP-148. In this system, covalently immobilized SAAP-148 retained bactericidal activity against Gram-positive, Gram-negative, and multidrug-resistant strains for up to 14 days in phosphate-buffered saline and remained active after prolonged exposure to diluted human plasma, whereas a physically embedded peptide lost activity within three days [103]. Importantly, immobilization significantly reduced cytotoxicity compared to a soluble peptide at comparable local concentrations, highlighting a key translational advantage of tethered systems [104].

Beyond sustained activity, covalent tethering enables precise control over peptide orientation and spacing—parameters that strongly influence antimicrobial efficacy. Studies on AMP-functionalized biomaterials demonstrate that peptide accessibility to bacterial membranes depends on tether length, flexibility, and exposure of cationic residues [103]. When improperly oriented or sterically constrained, immobilized peptides may bind bacteria without inducing membrane damage, underscoring the importance of rational integration design [101]. These findings are directly applicable to hydrogel systems, where network architecture and functional group distribution dictate how tethered peptides interact with cells and biofilms.

Stimuli-responsive hydrogels represent a further evolution in AMP delivery by coupling peptide release to pathological signals characteristic of infected tissues. Chronic wounds and biofilm-associated infections are marked by elevated reactive oxygen species (ROS), increased matrix metalloproteinase (MMP) activity, acidic pH, and inflammatory enzymes, all of which can be exploited as endogenous triggers for hydrogel degradation or bond cleavage [105,108]. ROS-responsive hydrogels incorporating boronate ester or thioether linkages undergo accelerated degradation under oxidative stress, enabling selective AMP release in infected environments while remaining stable under normal physiological conditions [106,107]. Similarly, MMP-cleavable peptide crosslinkers allow hydrogels to degrade preferentially in protease-rich wound beds, synchronizing AMP availability with disease severity [108]. These infection-triggered systems reduce unnecessary peptide loss and minimize off-target exposure, addressing a major limitation of passive release platforms.

Hybrid integration strategies that combine covalently tethered AMPs with releasable peptide reservoirs offer a particularly promising route toward comprehensive antibiofilm control. In such systems, immobilized peptides provide immediate surface-mediated killing and inhibit bacterial adhesion, while entrapped or stimuli-released peptides diffuse into surrounding tissue and biofilm structures to suppress residual and planktonic bacteria [100]. This dual functionality mirrors approaches used in antimicrobial implant coatings, where contact-active surfaces are combined with localized release to prevent both initial colonization and long-term biofilm maturation [103]. Hybrid hydrogels are especially well suited for chronic wounds and indwelling devices, where fluctuating microbial burdens and prolonged exposure necessitate both rapid and sustained antimicrobial action [99,102].

Successful translation of AMP–hydrogel systems ultimately depend on careful application of hydrogel chemistry, peptide orientation, diffusion limitations, and the complex biofilm microenvironment. Crosslink density and mesh size govern peptide mobility and release kinetics, while hydrogel charge and hydrophilicity influence peptide–polymer interactions and microbial adhesion [98,100]. Biofilms present additional challenges, including dense EPS, local pH gradients, limited diffusion, and high protease activity, all of which can attenuate AMP efficacy if not accounted for during design [101]. Consequently, integration strategies must be tailored to the intended clinical context, balancing peptide stability, bioavailability, and manufacturability to achieve durable antibiofilm performance.

Building on these integration and release strategies, the next section focuses on how AMP–hydrogel design translates into measurable antibiofilm activity, including the underlying mechanisms of biofilm disruption and the experimental models used for in vitro, ex vivo, and in vivo evaluation.

## 6. Multifunctional Systems with Antibiofilm Properties

Antimicrobial materials, particularly hydrogels, are being developed to address chronic infections. Hydrogels are highly hydrated 3D porous materials, which consist of polymer chains with either physical or chemical crosslinking [109,110] that can be functionalized for antibacterial, tissue-regenerating, and drug-delivery applications [111]. Composite hydrogels can covalently or physically incorporate a wide range of antimicrobial agents, including antibiotics, antimicrobial peptides, biological compounds, polysaccharides, and nanoparticles (NPs) to help combat bacterial resistance. The incorporation of these components, along with modifications to monomer composition and crosslinking density, enhances key properties of composite antibacterial hydrogels, such as hydrophilicity and porosity [112].

**Figure 5:**
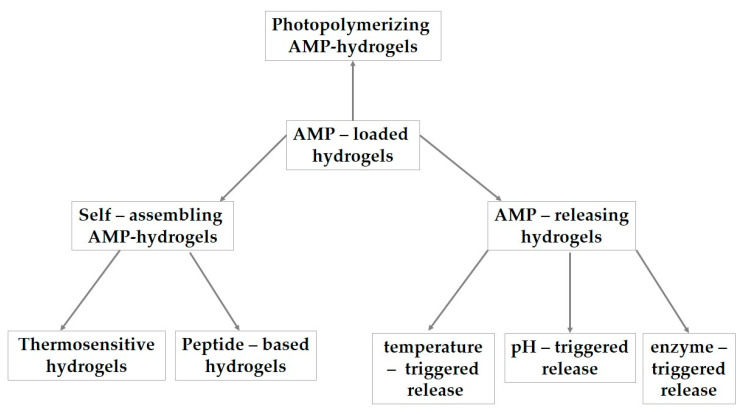
Multifunctional hydrogels functionalized with AMPs.

Hydrogels are commonly synthesized by rapid photopolymerization under visible or UV light using photoinitiators, but there are also self-assembling AMP–hydrogels and AMP-releasing hydrogels [100] as shown in Figure 5.

### 6.1. Photoplymerizing AMP–Hydrogels

Photopolymerization enabling precise functionalization with thiolated molecules such as peptides [113]. Using this approach, antimicrobial peptide AMP-loaded hydrogels have been developed by conjugating norbornene-modified chitosan with thiolated Dhvar5 AMP–hydrogels that significantly reduced *Staphylococcus epidermidis* adhesion and enhanced *P. aeruginosa* killing while remaining biocompatible [114,115]. Similarly, battacin lipopeptide hydrogels were synthesized with strong antibacterial and antibiofilm activity against *P. aeruginosa* and *S. aureus*, excellent cytocompatibility, and no hemolytic effects [116]. These properties highlight the potential of AMP-based hydrogels for clinical applications [100].

Hydrogels were actively developed for wound dressing applications by integrating AMPs and inorganic components to enhance antibacterial activity and healing. Thus, a sprayable gelatin methacrylate (GelMA)-based hydrogel incorporating dopamine, cerium oxide nanoparticles (CeONs), and the AMP HHC-36 (KRWWKWWRR) was developed [117]. These hydrogels exhibited near-complete bactericidal activity against both Gram-positive and Gram-negative pathogens [118], promoted accelerated wound healing and skin regeneration in mouse models, and enhanced collagen deposition [119].

Although hydrogels are highly biocompatible and can mimic the extracellular matrix [120], they often suffer from poor mechanical strength, wet-tissue adhesion, and limited antimicrobial function [121]. To address these limitations, a hydrogel based on GelMA and methacrylated tropoelastin (MeTro), functionalized with the AMP Tet 213 via visible-light crosslinking was obtained [122]. These hydrogels showed enhanced adhesive and sealing performance, effective inhibition of MRSA and *E. coli*, support for fibroblast growth, minimal inflammation, and efficient biodegradation in vivo, making them strong candidates for advanced wound-closure applications [123].

Liu and coworkers developed AMP-loaded hydrogel coatings with combined antibacterial, antithrombotic, and antifouling properties for medical device applications. The coatings based on sulfobetaine methacrylate and acrylic acid hydrogels grafted onto polyvinyl chloride surfaces and functionalized with AMPs WR (WRWRWR-NH2) or Bac2A (RLARIVVIRVAR-NH2) showed excellent hemocompatibility, completely inhibited *E. coli* and *S. aureus* surface growth, and retained antifouling and antithrombotic performance. These hydrogels demonstrated superior long-term antimicrobial stability and effectively treated catheter-associated *S. aureus* infections in a rat model, highlighting their potential as antimicrobial coatings for blood-contacting medical devices [124].

### 6.2. Self-Assembling AMP–Hydrogels

Chronic wounds represent a massive burden on healthcare systems around the globe, due to major complications. Thus, hydrogels based on self-assembling polypeptides have emerged as promising materials by acting simultaneously as a scaffold for skin cell proliferation and as a delivery system for therapeutic molecules [125]. The most studied self-assembling AMP–hydrogels are the thermosensitive hydrogels and the peptide-based self-assembling hydrogels [100].

Self-assembling hydrogels offer advantages over photopolymerizable systems because they form without light, enabling minimally invasive therapeutic delivery and use in situations where light is not available [126]. Their assembly can be triggered by stimuli such as ionic strength, pH, or temperature, undergoing sol–gel transitions around critical solution temperatures [127]. For example, a thermosensitive poly (N-isopropyl acrylamide) (PNIPAM)-based self-assembling hydrogel incorporating a joint peptide composed of the AMP (RRWRVIVKW) and the self-assembling peptide RADA16 (RA-Amps) to promote wound healing was developed. These hydrogels rapidly assembled at physiological temperature, showed strong in vitro bactericidal activity against *E. coli* and *S. aureus*, and improved epithelialization, angiogenesis, and collagen formation in a murine wound model, although in vivo antimicrobial efficacy was not directly demonstrated [127].

Self-assembling hydrogels can be entirely peptide-based, forming three-dimensional fibrous networks through noncovalent interactions such as ionic bonding, hydrophobic forces, hydrogen bonding, and π–π stacking [128]. Thus, FKF (Phe–Lys–Phe) peptide hydrogels that rapidly self-assembled under acidic conditions were developed, exhibiting broad-spectrum in vitro antibacterial activity and moderate in vivo efficacy, though wound closure was delayed, likely due to acidic polymerization conditions [129].

To improve biocompatibility, one study synthesized a D-amino-acid version of the self-assembling peptide KLVFFAK (KKd-11), which rapidly formed hydrogels at neutral to basic pH and effectively inhibited and eradicated *E. coli* and *S. aureus* biofilms, having proteolytic stability and biocompatibility. Due to these properties, KKd-11 hydrogels show strong potential for preventing wound infections and bacterial colonization on medical devices [130].

A self-assembling antimicrobial peptide hydrogel based on the amphipathic hexapeptide PAF26 (Ac-RKKWFW-NH_2_) was developed, forming polymerized structures at physiological pH and exerting antimicrobial activity through permeabilization of microbial cell membranes. The resulting hydrogels showed strong in vitro efficacy, completely inhibiting the growth of *Candida albicans*, *S. aureus*, and *E coli*. While these properties indicate strong clinical potential, further optimization is needed, as cytotoxicity of the PAF26 hydrogels was not evaluated [131].

In another study, a self-assembling antibacterial lipopeptide hydrogel was developed based on palmitic acid-conjugated NAVSIQKKK (PA-NV), which effectively inhibited *E. coli* and *S. aureus* at moderate bacterial loads, while higher inocula reduced efficacy. These PA-NV hydrogels were biocompatible, non-cytotoxic to mammalian cells, and resistant to enzymatic degradation [132].

Self-forming antimicrobial peptide (AMP) hydrogels can be enhanced by incorporating additional bioactive networks to support wound healing. Thus, hyaluronic acid (HA) has been combined with AMP KK(SLKL)_3_KK to form AMP–HA that showed strong in vitro antibacterial activity against *Staphylococcus aureus* and *E. coli*. In vivo, AMP–HA hydrogels significantly accelerated wound closure and promoted collagen deposition, epithelialization, and angiogenesis in a mouse model, highlighting their potential as multifunctional antimicrobial wound-healing materials for clinical use [133].

Self-assembling RADA16 hydrogels incorporating the antimicrobial peptide Tet213 have been developed, providing sustained peptide release over 28 days and effectively reducing *S. aureus* growth in a concentration-dependent manner. In a rabbit osteomyelitis model, RADA–AMP–hydrogels limited bacterial-induced inflammation and promoted new bone formation, suggesting strong potential for treating bone infections and supporting bone repair [134].

A self-assembling hydrogel combining the amphiphilic AMP Jelleine-1 (J1, PFKLSLHL-NH_2_) with adenosine diphosphate (ADP) demonstrated in vitro antimicrobial activity against *S.aureus*, *E. coli*, *C. albicans*, and MRSA strains. In a mouse model, the hydrogel reduced postoperative intraperitoneal adhesions and promoted efficient hemostasis through enhanced blood coagulation, platelet activation, and adhesion. Its injectable nature makes it suitable for surgical applications over large areas prone to adhesions [135,136].

Methylcellulose hydrogel loaded with AMP D-Bac8c2,5 Leu reduced biofilm viability in both monospecies and polymicrobial biofilms of *S. aureus* and *P. aeruginosa* [137].

### 6.3. AMP-Releasing Hydrogels

The use of nanomaterials for therapeutic delivery enables precise spatiotemporal control of payload release, improving efficacy while minimizing off-target effects and cytotoxicity. This controlled-release approach is being applied to antimicrobial peptide (AMP) delivery, with current strategies focusing on temperature, pH-, and enzyme-triggered release mechanisms [138].

Thermo-responsive chitosan (TCTS) hydrogels as controlled-release carriers for the AMP piscidin-1 were developed [139,140]. TCTS hydrogels exhibited strong bactericidal activity against both standard and drug-resistant *Acinetobacter baumannii*, with 40% AMP released on day 1 and sustained release up to 7 days, achieving 90% cumulative release, while remaining non-cytotoxic to human fibroblasts [141].

Extending temperature-controlled release, a light-activated, photothermal AMP–hydrogel was developed by encapsulating the AMP IK8 in liposomes and embedding them with gold nanorods (AuNRs) in a PEG-based hydrogel. Near-infrared irradiation (860 nm) triggered AMP and AuNR release, achieving ~65% release at 55 °C, resulting in significant killing of *P. aeruginosa* and *S. aureus* without cytotoxicity. The inclusion of AuNRs enhanced photothermal effects and bactericidal activity, particularly against biofilms, but in vivo studies and further optimization for stability and biocompatibility are required [142,143].

A degradable hydrogel was constructed with gelatin methacrylate (GelMA), tannic acid (TA), and polyphosphate (PolyP) by photopolymerization, and mesoporous polydopamine nanoparticles (MPDA) with berberine (BR), along with Cu and Bi nanoparticles, having high photothermal conversion efficiency (68.3%) and antibiofilm properties. Thus, 99.34% of methicillin-resistant *S. aureus* (MRSA) and 99.82% of *E. coli* were killed in vitro [144].

On the other hand, pH-sensitive hydrogels provide controlled AMP release in response to the acidic environment of infected wounds, improving antimicrobial efficacy and wound healing. Several DP7-ODEX hydrogels, containing the cationic AMP DP7 and oxidized dextran, which degrade under acidic conditions to release the AMP were developed. These hydrogels demonstrated broad-spectrum antimicrobial activity against *P. aeruginosa*, *S. aureus*, and *E. coli*, with enhanced efficacy when combined with ceftazidime against multidrug-resistant strains. In normal and diabetic mouse models, DP7-ODEX and CAZ-DP7-ODEX hydrogels accelerated wound closure, promoted dermal and epidermal regeneration, and were well tolerated, though further studies are needed to confirm long-term safety and generalizability [145,146].

Similarly, pH-sensitive ODEX/HA-AMP–hydrogels were designed by incorporating the AMP cecropin and platelet-rich plasma (PRP) to both reduce bacterial burden and enhance wound healing. These hydrogels released AMPs steadily over 14 days and provided controlled release of growth factors (PDGF, TGF-β, EGF), promoting collagen deposition, angiogenesis, and skin regeneration in a diabetic mouse wound model [147,148,149].

Enzymatic cleavage-triggered AMP release is a third strategy for controlled antimicrobial delivery. DNA-based hydrogels were previously developed and loaded with the cationic AMP L12, which bind polyanionic DNA nanostructures and release AMPs in response to bacterial DNase activity [150]. L12-loaded hydrogels showed strong activity against methicillin-resistant and susceptible *S. aureus* and *E. coli*, with release rates dependent on DNase concentration. Ex vivo porcine skin studies demonstrated a 4-log reduction in *S. aureus* within 4 h, while in vivo murine wound models exhibited faster wound closure, increased granulation tissue, and anti-inflammatory effects through TNF-α reduction. These hydrogels are promising for deep or chronic wound treatment, though further optimization is needed to improve AMP retention [151].

Similarly, in another study, an infection-responsive hydrogel was developed for diabetic wounds by conjugating the AMP KR12 to adamantane-modified HA using a cyclic linker cleavable by reactive oxygen species (ROS) and matrix metalloproteases (MMPs). This design enabled AMP release only in infected environments, minimizing toxicity while maintaining antimicrobial activity against *E. coli* and *S. aureus*. The hydrogel supported fibroblast proliferation in vitro and accelerated wound healing in an infected diabetic mouse model. These enzymatically responsive supramolecular hydrogels offer controlled AMP release, effective infection treatment, and enhanced wound healing, making them highly promising for chronic wound management, especially in diabetic patients [152,153].

## 7. Conclusions and Perspectives

Urgent development of novel treatment approaches is essential to combat multidrug-resistant pathogens and avert ongoing chronic wound infections. Research on peptides as medicinal agents has significantly increased over the years due to their properties. Engineered hydrogels containing synthetic peptides are set to revolutionize antimicrobial therapy. Upcoming studies are anticipated to focus on logical peptide formulation, improved resistance to enzymatic breakdown, and the creation of multifunctional, responsive hydrogels that facilitate accurate, on-demand antimicrobial release.

Extensive application of peptides is still constrained, partly because of their vulnerability to enzymatic breakdown. The use of conformational restraints, non-natural amino acids, peptide bond mimics, or non-peptidic scaffolds can greatly enhance their stability and bioactivity.

In addition to enhancing enzymatic stability, utilizing various chemical modifications along with suitable delivery methods could potentially produce considerably better peptides. In silico techniques, including machine learning and deep learning, provide powerful tools for guiding peptide design by predicting sequence–property relationships and prioritizing candidates with potential for improved bioactivity and stability. However, their role is supportive, as the actual enhancement of peptide performance ultimately depends on experimental validation and optimization.

To conclude, the integration of synthetic antimicrobial peptides with designed hydrogels is anticipated to recast future antimicrobial treatments. Future advancements will concentrate to enhance peptide stability, minimize cytotoxicity, and create hydrogels featuring controlled-release and stimuli-responsive properties. This method shows significant promise for tackling antimicrobial resistance, wound healing, and facilitating personalized, localized infection control.

## Figures and Tables

**Table 3:** Hydrogel integration strategies for AMPs delivery.

Integration Strategy	Mode of AMP Presentation	Antibacterial Mechanism	Stability and Durability Outcomes	Key Advantages	Main Limitations and Design Risks	References
Physical entrapment	AMPs freely embedded within hydrogel mesh and released by diffusion and/or matrix degradation	Diffusion-driven killing of planktonic bacteria and early-stage biofilms	Antibacterial activity decreases over time due to peptide leaching and proteolytic degradation in wound fluid	Simple fabrication. Compatible with many natural and synthetic hydrogels. Enables sustained local exposure	Burst release during early swelling. Susceptibility of free peptides to host- and bacteria-derived proteases. Limited durability against mature biofilms.	[99,100,101]
Hybrid tethered + releasable systems	Combination of immobilized AMPs at the hydrogel surface and diffusible AMPs within the network	Immediate contact-killing at material interface plus sustained diffusion-mediated antibiofilm activity	Short-term surface protection combined with longer-term suppression of residual and planktonic bacteria	Broad-spectrum antibiofilm coverage; prevention of initial colonization and long-term biofilm maturation.	Increased formulation complexity. The need to balance loading density, release kinetics, and host compatibility.	[99,100,102]
Covalent tethering (contact-active hydrogels)	AMPs covalently immobilized on hydrogel backbone or surface via stable chemical linkages	Contact-mediated membrane disruption upon bacterial attachment (“contact-killing”)	Prolonged antibacterial activity due to prevention of peptide leaching; immobilized AMPs remain active under prolonged aqueous and plasma exposure	Strong localization of activity. Reduced systemic exposure. Improved peptide stability. Reduced cytotoxicity compared to soluble peptides.	Antibacterial efficacy depends on peptide orientation, density, and spacer length. Limited penetration into deep biofilm layers without releasable fraction.	[103,104]
Stimuli-responsive (infection-triggered) release	AMPs entrapped or linked via cleavable bonds responsive to pathological cues (ROS, MMPs, pH)	Triggered release aligned with infection severity, enhancing killing under pathological conditions	Stable under physiological conditions; accelerated degradation and AMP release in infected or inflamed microenvironments	On-demand peptide availability. Improved efficacy with minimized off-target exposure. Better synchronization with disease state.	Variability in trigger intensity between patients and wound types. Complexity of validating clinically relevant stimulus thresholds.	[105,106,107]
Design constraints across strategies	AMP behavior governed by hydrogel chemistry, crosslink density, mesh size, charge, and peptide orientation	Modulation of diffusion, accessibility to bacterial membranes, and resistance to biofilm EPS barriers	Performance depends on matching hydrogel degradation and peptide stability to biofilm microenvironment	Enables rational optimization for specific clinical contexts (wounds vs. implants).	Trade-offs between mechanical properties. Diffusion limits. Peptide stability, and manufacturability.	[98,101,103]

The effects summarized represent general trends reported across multiple studies; antibacterial performance, stability, and durability are highly context-dependent and influenced by peptide sequence, hydrogel composition, and experimental model. AMP, antimicrobial peptide; EPS, extracellular polymeric substance; MMP, matrix metalloproteinase; ROS, reactive oxygen species.

## Data Availability

No additional data are available.

## Abbreviations

The following abbreviations are used in this manuscript:

ADP	Adenosine diphosphate
AFPs	Antifungal peptides
AMPs	Antimicrobial peptides
AuNRs	Gold nanorods
BaAMPs	Biofilm-active antimicrobial peptides database
BR	Berberine
CeONs	Cerium oxide nanoparticles
CFU	Colony-forming units
CLSM	Confocal laser scanning microscopy
CV	Crystal violet
DNA	Deoxyribonucleic acid
EGF	Epidermal growth factor
EPS	Extracellular polymeric substances
GelMA	Gelatin methacrylate
GFP	Green fluorescent protein
HA	Hyaluronic acid
MBEC	Minimum biofilm eradication concentration
MBIC	Minimum biofilm inhibitory concentration
MIC	Minimum inhibitory concentration
MMP	Matrix metalloproteinase
MMPs	Matrix metalloproteinases
MPDA	Mesoporous polydopamine nanoparticles
MRSA	Methicillin-resistant *Staphylococcus aureus*
NPs	Nanoparticles
OD	Optical density
ODEX	Oxidized dextran
PBS	Phosphate-buffered saline
PDGF	Platelet-derived growth factor
PEG	Polyethylene glycol
PEGylation	Poly(ethylene glycol) conjugation
PNIPAM	Poly(N-isopropyl acrylamide)
PolyP	Polyphosphate
ppGpp	Guanosine tetraphosphate
pppGpp	Guanosine pentaphosphate
PRP	Platelet-rich plasma
RNA	Ribonucleic acid
ROS	Reactive oxygen species
TA	Tannic acid
TCTS	Thermo-responsive chitosan
TGF-β	Transforming growth factor beta
TNF-α	Tumor necrosis factor alpha
UV	Ultraviolet
